# Has the short-term effect of black smoke exposure on pneumonia mortality been underestimated because hospitalisation is ignored: findings from a case-crossover study

**DOI:** 10.1186/1476-069X-12-97

**Published:** 2013-11-07

**Authors:** Matthew Gittins, Roseanne McNamee, Melanie Carder, Iain Beverland, Raymond M Agius

**Affiliations:** 1 Biostatistics Group, University of Manchester, Jean McFarlane Building, Oxford Road, Manchester M13 9PL, England, UK; 2 Centre for Occupational and Environmental Health, The University of Manchester, Humanities Building, Oxford Road, Manchester M13 9PL, England, UK; 3 Department of Civil Engineering, University of Strathclyde, John Anderson Building, 107 Rottenrow, Glasgow G4 ONG, Scotland, UK

**Keywords:** Air pollution, Time-stratified, Case-crossover, Community, Lag stratified, Lag distributed

## Abstract

**Background:**

Short-term associations have been demonstrated between air pollution and respiratory mortality including pneumonia. Studies typically estimate exposure based only on place of residence, yet many are in hospital prior to death. This study investigates lag length and tests the hypothesis that the effect of 'black smoke’ is greater when restricted to pneumonia deaths in the community – Community Deaths from Pneumonia.

**Methods:**

A time-stratified case-crossover design using conditional logistic regression estimated the daily percentage increase in risk of pneumonia mortality in relation to 'black smoke’ in the preceding 30 days. Cases were pneumonia deaths in Edinburgh 1981–1996. Multiple 'control’ periods, were defined using the same weekdays for the same month as the case death. Lag structure was investigated by a stratified lag model with five 6-day periods and by distributed lag models. Hospital admissions data, defined a community death as someone who had not been in hospital in any of the 30 days before death.

**Results:**

Of 14,346 subjects who died from pneumonia, 7,536 were community deaths. Larger estimated increases in risks were seen in the community for all lag periods. Both stratified and distributed lag methods suggested positive effect estimates for 18 days after exposure and negative thereafter; the average percent increase per day across the 18 days was 0.70% (95% C.I. 0.29-1.14) for community subjects and 0.30% (95% C.I. 0.03-0.59) for all subjects.

**Conclusions:**

Studies which fail to account for hospitalisation may underestimate exposure effects as stronger pollution effects on mortality were evident in community based subjects.

## Background

Many studies worldwide have demonstrated an association between air pollution and all cause mortality [1,2], specifically respiratory mortality [3-5]. In 2011, pneumonia was the 6^th^ leading cause of death in England and Wales for males (10,824) and 4^th^ (14,872) for females [6]. However, only a few studies - with limited findings - have specifically investigated associations between pneumonia related deaths and ambient air pollution. Schwartz and Dockery, indicated an increase in pneumonia mortality of 11% (95% C.I. -3%, 27%) per 100 μgm^-3^ increase of Total Suspended Particles (TSP) [7]. Halonen et al. demonstrated a percentage increase in pneumonia mortality in Finland of 3.16% (95% C.I. -2.64%, 9.32%) per increase in inter-quartile range of a 5 day Coarse Particle Matter (PM_10-2.5_) mean [8]. Zanobetti et al. proposed that air pollution may be a predisposing factor to Community Acquired Pneumonia (CAP) and that subjects with CAP rather than Hospital Acquired Pneumonia, may be more susceptible to the effects of air pollution [9]. Studies such as Neupane et al. have indicated a relationship between long-term exposure to air pollution and emergency visits to hospital with Community Acquired Pneumonia [10]. However, so far no study has investigated the effect of pollution on deaths from Community Acquired Pneumonia only.

Much of the evidence for the association between air pollution and general mortality has focused on exposure in a short time period – less than 40 days prior to death [2,11]. This focus on short to medium exposure may be appropriate for pneumonia which is generally an acute condition, though often associated with chronic underlying lung disease. Frequently, a deceased individual’s exposure is inferred from information regarding their place of residence with little or no attempt to take account of subject’s actual location, circumstances or activities. Exposure is typically assumed to be the same for all subjects living within a given distance from a single pollution monitor or an average of multiple monitors within the area [1,11]. More recent studies, trying to improve exposure estimates, have taken into account traffic density and other geographical information regarding the subjects neighbourhood or city [12,13]. The presumption still remained that the deceased was in the geographical location of residence during the exposure period; in fact it is common for people to die in non-residential locations (65.3% in a NHS hospital/Hospice in England and Wales [14]). If the hospital is located close to the place of residence, one might reasonably suppose that a patients’ exposure to outdoor pollution might be reduced when confined indoors [15]. Epidemiological observations have shown that deaths associated with air pollution, specifically TSP and Particle Matter with a diameter less than 10 μm (PM_10_), are disproportionately increased outside of hospital [16,17]. In addition, Jansen et al. 2002, found that the health effects of PM_10_ on cardiovascular disease and COPD in 14 U.S. cities decreased significantly as the proportion of homes with air conditioning increased [18]. Previous attempts at comparing risks in and out of hospital, such as Téllez-Rojo et al. 2001 and Zeka et al. 2006, have shown significant increased risk of death from respiratory or cardiovascular causes, in some cases up to a threefold increase. These studies have primarily used location at time of death without confirming location during exposure [19,20]. Failure to take account of hospitalisation during exposure could lead to further effect underestimation if a substantial fraction of the population experience reduced exposure in air-conditioned hospitals. A large proportion of subjects hospitalised during exposure might explain why some observational epidemiology studies based on routinely collected data may struggle to replicate previously demonstrated associations between pollution and pneumonia caused mortality [21].

Pneumonia occurs usually as a result of bacterial or viral infection. Often progressing rapidly within 24 hours, it presents symptoms such as coughing, chest pain, shortness of breath, and fever. Pneumonia can generally be diagnosed reliably through medical consultation and a chest radiography [22]. The relatively quick onset of the disease, short diagnosis period, and the time varying nature of air pollution exposure satisfies the conditions for employing the case-crossover design [23]. This design offers protection against possible subject level confounders without the need for complex modelling of mortality levels over time.

This study investigated the effect of 'black smoke’ (BS) over 30 days prior to pneumonia mortality using a time-stratified case-crossover design [24]. We tested the primary hypothesis that estimated association would be greater in subjects who spent the exposure period in the 'community’ (i.e. not in hospital) compared to those who spent some or all of the period in hospital. Members of the former group will be defined as subjects with a Community Death from Pneumonia (CDPs) which should be distinguished from CAP; CAP refers to a clinical category based on the source of pneumonia, CDP are a subgroup of CAP. Concurrently, hypothesis generating analyses explored how exposure affects mortality from pneumonia across the lag period. Subsequently, analyses based on the lag periods which showed effects on mortality were repeated for subgroups defined by gender and age. It is conceivable that differences in lifestyle between these groups could influence exposure or that there is increased susceptibility in older age, leading to differential observed effects of BS on CDPs.

## Methods

Deaths due to pneumonia (ICD-9 codes 480–487 pre 2000) between January 1981 and December 1996 from Edinburgh, Scotland formed the cases. Separately, information was provided on all admissions to hospital caused by respiratory, cardiovascular, lung cancer, diabetes, and digestive related causes for the same time period and location. The Information Services Division of NHS National Services in Scotland provided both datasets, they included; date of death, age, gender, admission dates, primary and secondary cause of admission and death, and if the patient died in hospital. The two files – one of deaths and one of hospital admissions – were linked to determine if the subject had been in hospital during the 30 days prior to death. Community Acquired Pneumonia refers to those subjects who did not acquire pneumonia from a hospital. Subjects with CAP may subsequently enter and later die in hospital. These subjects will not be included in our data as they will have had at least one day in hospital altering their exposure and increasing their chances of a secondary Hospital Acquired Pneumonia infection. A death was considered to be a Community Death from Pneumonia if the hospital admission data showed that the subject had not been in hospital for any of the 30 days prior to death. A CDP subject is therefore a special subgroup of the CAP deaths.

Daily black smoke air pollution data was obtained from one centrally located background monitoring site and hourly ambient air temperature (between 7 am-11 pm) was provided by the Scottish Meteorological Office. This was used to give daytime mean temperature and mean pollution levels for the area. For each date of death, or case day, 'black smoke’ daily results for the month prior were averaged firstly across 1–30 days and then separately for 1–6, 7–12, 13–18, 19–24, and 25–30 days. These formed the exposure variables for the cases. Same day exposure (lag 0) was not included as pneumonia has a minimum 24 hr incubation period [22], and same day exposure potentially includes exposure after death. Temperature displays temporal associations with pollution levels, and has previously been shown in this population to have an approximately double linear relationship with mortality, with a knot at 11°C [25]. Hence, two continuous temperature variables were calculated as, “high” (t-11 if ≥11°C, 0 otherwise) and “low” (t-11 if ≤11°C, 0 otherwise), where t is the daytime mean temperature. Average temperature across lags 1–30 days for both “low” and “high” variables was included in all models. Including pollution and temperature exposure lags (up to 30 days prior) reduces the chances of underestimating the exposure effect [11]. Further information regarding the data source and variable manipulations can be found elsewhere [25].

The 30 days prior to the date of death from pneumonia defined the case exposure period. In case-crossover designs, reduced bias allows for a time-stratified design to select matched control periods [26]. Control days were defined as all equivalent days of the week within the same month as the case day, in order to account for any weather, seasonal, or day of the week confounding [27]. For example, if the event occurred on the second Monday of May then the control days became all other Mondays of May. The control exposure period was then 30 days prior to the control day, and temperature and pollution variables were formed from these periods. Ethical approval for the study was granted by the NHS Research Ethics Committee (Ref – 11/NW/0768) and the Privacy Advisory Committee associated with the Information Services Division Scotland once appropriate measures had been implemented to ensure any participant information was securely stored.

### Statistical analysis

Conditional logistic regression [28], initially compared average exposure between the case and controls over 30 days prior to death (Model 1). To investigate exposure during the 30 days, a lag-stratified model fitted five exposure variables each representing a 6 day lag period (Model 2). To avoid problems comparing estimates based on different period lengths (e.g. 30 days and 6 days) [25,29], all estimated coefficient were divided by the number of days on which the mean was based. Hence, BS results are expressed as percentage increase in risk associated with an increase of 10 μgm^-3^ ((e^B*10^-1)*100, where B = model coefficient) on any individual day within the lag period. Similarly, both continuous temperature variables are also expressed as a percentage increase in risk corresponding to a *decrease in 1°C of temperature* for any individual day during lag period ((e^-B^-1)*100). The association between BS and All Pneumonia deaths (AP) was estimated before restricting to the subgroup (CDP) deemed to have the greatest potential exposure. To test if a significant difference in exposure effects occurred, an interaction term was included to compare CDP and non-CDP for each BS lag term, and the Log-likelihood-ratio was used to test the difference.

Within each lag period the average daily value across the lag period was calculated so long as a minimum of four out of every six days contained a pollution estimate. The analysis was then performed with any subject with complete data for the case day and at least one control day. This resulted in 4.5% and 4.3% (AP and CDP, respectively) of subjects dropped due to either missing pollution data in the case day or all control days*.*

The effect of a change in exposure on an individual day might be expected to vary across subsequent days and eventually fall to zero [30]. An *estimated* effect might even become negative afterwards if the 'high risk’ pool of subjects is depleted without sufficient replenishment, causing a mortality rate lower than the underlying rate [17]. In addition to lag-stratified, a quadratic distributed lag [30] estimated the lag time, L, before which the estimated effects are positive. For simplicity in further analysis, average exposure across the period (0,L) days was modelled for each gender and two age groups (≤80 and >80). Analysis was performed using STATA version 11 [31].

## Results

A total of 15,784 people had pneumonia as primary or secondary cause of death in Edinburgh between 1981 and 1996. However, missing pollution data (9.8% of days during the time period) for either a subject’s case day or all of the subject’s control days meant only 14,346 cases with 47,431 control days were eligible for the analysis. Table 1 gives descriptive statistics for the daily average BS air pollution, air temperature and demographic characteristics for both all pneumonia (AP) and CDP (52.5% of the AP) subject groups. Further, summary statistics indicated an inter-quartile range approximately 10 μgm^-3^ for each lag period.

**Table 1:** Descriptive statistics of exposure data and study subjects split by all pneumonia and community deaths from pneumonia

	**Mean**	**S.D.**	**Median**	**IQR**	**Min**	**Max**
Daily Ave Air Temp (°C)	9.4	5.1	9.4	8	-12.7	24.48
Daily Ave BS (μgm^-3^)	12.7	13.3	9	10	1	194
Lag 1–6 days Ave BS (μgm^-3^)	12.9	10.8	9.3	9.5	1	95.2
Lag 7–12 days Ave BS (μgm^-3^)	13	11.1	9.5	9.6	1	95.2
Lag 13–18 days Ave BS (μgm^-3^)	13.1	11.3	9.5	9.7	1	95.2
Lag 19–24 days Ave BS (μgm^-3^)	13.1	11.2	9.5	9.5	1	95.2
Lag 25–30 days Ave BS (μgm^-3^)	13	11.2	9.5	9.3	1	95.2
Lag 1–30 days Ave BS (μgm^-3^)	13.2	9.2	10.1	9.3	2.7	73.2
Age (CDP)	79.13	12.6	82	12	0	108
Age (Non-CDP)	79.21	11.92	81	13	0	108
Age (AP)	79.15	12.2	81	13	0	108
	Gender		Age Grouped			
Categories	Male	Female	<80	≥80		Total
CDP only Subjects (%)	3,409 (45.2)	4,127 (54.8)	3,064 (40.7)	4,472 (59.3)		7,536 (100)
Non-CDP only Subjects (%)	3,166 (48.2)	3,644 (46.9)	3,109 (50.4)	3,701 (45.3)		6,810 (47.5)
All Pneumonia Subjects (%)	6,575 (45.8)	7,771 (54.2)	6,173 (43.0)	8,173 (57.0)		14,346 (100)

BS = Black Smoke.

The percentage change in relative risk (%RR), with 95% confidence intervals, are given in Table 2 for an increase in BS of 10 μgm^-3^ or a *temperature decrease* of 1°C on any individual day. MODEL 1 and 2 refers to the model with BS and temperature averaged over 1–30 days and BS split into five smaller lag periods, respectively. Correlation coefficients indicate strong correlation between average exposures in adjacent lag periods (≈0.7) however the corresponding variance inflation factors (VIF = 2.01 to 2.78) did not indicate the presence of collinearity between the five lag periods in model 2 [32]. To easily compare effect sizes between lag periods the percentage change in relative risk corresponds to the effect of a change in BS or temperature on any individual day within the associated lag period. The differences in %RR between AP and CDP along with corresponding significance levels are also given.

**Table 2:** Percent change in risk for lagged black smoke air pollution and pneumonia mortality: repeated for AP (All pneumonia), CDP (Community death from pneumonia) & Non-CDP

		**AP**		**CDP only**		**Non - CDP only**				
	**Lag (days)**	**% RR change**	**95% C.I.**	**% RR change**	**95% C.I.**	**% RR change**	**95% C.I.**	**|CDP|-|AP| diff**	**|CDP-Non CDP| diff**	**P-val**
MODEL 1 Black Smoke	1-30	0.08%	-0.17%, 0.35%	0.19%	-0.16%, 0.58%	0.01%	-0.34%, 0.40%	0.11%	0.18%	0.285
Air Temp “Low”	1-30	0.20%	0.11%, 0.29%	0.22%	0.09%, 0.35%	0.16%	0.03%, 0.30%	0.02%	0.06%	0.502
Air Temp “High”	1-30	-0.05%	-0.20%, 0.10%	-0.20%	-0.40%, 0.00%	0.11%	-0.11%, 0.34%	0.15%	0.31%	0.059
MODEL 2 Black Smoke	1-6	0.12%	-0.37%, 0.62%	0.56%	-0.14%, 1.29%	-0.31%	-0.99%, 0.41%	0.44%	0.87%	0.022
	7-12	0.05%	-0.42%, 0.53%	0.32%	-0.33%, 1.00%	-0.25%	-0.92%, 0.45%	0.28%	0.57%	0.023
	13-18	0.40%	-0.08%, 0.90%	0.71%	0.03%, 1.42%	0.14%	-0.56%, 0.86%	0.31%	0.57%	0.163
	19-24	-0.09%	-0.55%, 0.38%	-0.16%	-0.79%, 0.50%	0.05%	-0.63%, 0.75%	0.06%	0.21%	0.272
	25-30	-0.11%	-0.57%, 0.36%	-0.39%	-1.01%, 0.25%	0.34%	-0.35%, 1.06%	0.28%	0.73%	0.008
Air Temp “Low”	1-30	0.19%	0.10%, 0.29%	0.19%	0.06%, 0.32%	0.18%	0.05%, 0.32%	0.00%	0.01%	0.487
Air Temp “High”	1-30	-0.05%	-0.19%, 0.10%	-0.20%	-0.39%, 0.01%	0.09%	-0.12%, 0.33%	0.15%	0.29%	0.058

%RR Change - percentage change in Relative Risk, associated with an increase of 10 μgm^-3^ BS or a decrease of 1°C, on any individual day within the lag period, with corresponding 95% Confidence Interval (95% C.I.).

Model 1 - One 30 day lag, Model 2 - The 30 days split into 5 lags of 6 days each fitted simultaneously.

CDP – AP Diff - The difference in the magnitude of the effect size between AP and CDP (CDP-AP).

CDP-Non CDP Diff - The difference in the effect size between CDP and Non-CDP (CDP – Non CDP).

**Figure 1:**
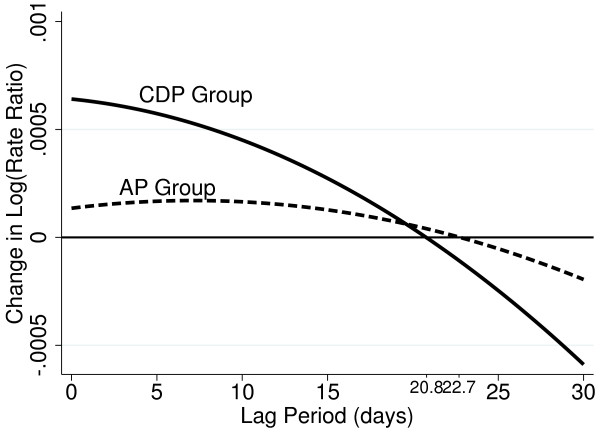
The quadratic lag distribution model for subjects with all pneumonia and community deaths from pneumonia.

MODEL 1 considers the effects of exposure on each of the 30 days to be equal. An increase of 10 μgm^-3^ black smoke on any of the 30 days, showed a small rise in AP relative risk increasing to 0.19% in the CDP group, resulting from a %RR difference of -0.18% between CDP and non-CDP subjects (CDP-non-CDP %RR). When the 30 days is split into 5 lag periods (MODEL 2), the magnitude of the effect is always larger in the CDP subjects, of whom the largest changes in %RR are seen in the 1–6, 7–12, and 13–18 day lags. This 18 day period prior to death appeared to be the high risk period, as an increase%RR is observed in 1–6, 7–12, and 13–18 day lags whereas a decrease is observed in the 19–24 and 25–30 day lags. Figure 1 plots the change in log rate ratio associated with the 30 day lag period for both AP and CDP as modelled using the quadratic lag distribution model. As suggested in Table 2, the CDP group showed larger effects with a more rapid decline crossing zero at approximately 21 days, almost 2 days earlier than the more gradual AP decline in risk.

Low Temperature effects changed very little, a 1°C decrease corresponded to an increase in relative risk approximately the same for both AP and CDP. In comparison, a 1°C decrease in high temperature shows a small decrease in risk in AP that increases in magnitude in the CDP group.

A secondary analysis concentrated on the minimum lag period where increased risk was observed (18 days) and split the data into the subgroups; gender (male/female), and age (<80/≥80). In all but low temperature for males & age < 80 an increase in %RR was seen in the CDP group. The CDP subjects indicated a stronger %RR in males (0.83% - 95% C.I. 0.21%, 1.51%) compared to females (0.61% - 95% C.I. 0.08%, 1.19%). Subjects aged 80 or above showed larger relative risk in AP (0.37% - 95% C.I. 0.00%, 0.77%; to 0.23% - 95% C.I. -0.17%, 0.66% in <80) but the difference disappeared when restricted to CDP subjects (0.71% - 95% C.I. 0.18%, 1.30% and 0.71% - 95% C.I. 0.10%, 1.38%, respectively). Analysis was repeated for subjects aged less than 65 to investigate different age distributions of pneumonia subtypes. Even though the effect sizes showed slightly larger differences in magnitude compared to the less than 80 group they were highly imprecise due to substantially smaller sample size, hence they have not been reported here. Comparisons using the log-likelihood test were performed between the gender and age groups; in all cases the difference was non-significant at the 5% level. Further information can be found in the Additional file 1: Table S1.

## Discussion

These findings suggest that a subject’s location is an important consideration when assessing the effect of ambient air pollution on pneumonia mortality. In this study, the percentage relative risk of air pollution was significantly higher in CDP compared to AP by 0.44% (1–6 day lag), 0.28% (7–12 day lag) with a further important but non-significant increase of 0.31% in the 13–18 day lag. The 18 days prior to death indicated a lag period of increased risk, with the largest relative risk (CDP = 0.71% (0.03%, 1.42%)) in the 13–18 day lag period. Prior to the 18^th^ day a decrease in relative risk occurred, with the magnitude increasing in the lag period furthest from the event. The quadratic distributed lag plot confirmed an increased risk period of 20–22 days prior to the event. The decrease effect on mortality may be due to a small pool of people susceptible to dying from pollution effects [17]. Initial changes in pollution cause the pool to diminish more rapidly than it can be replenished, creating a net deficit in the number of susceptible subjects; this leaves a larger proportion of stronger subjects to experience the longer lag periods and hence, a reduction in deaths.

In all lag periods, irrespective of the direction, the magnitude of the effect is greater in the CDP group. Hence, significant associations between BS and pneumonia mortality may have been overlooked in previous studies that have not taken into account location during exposure. Removing those who may have had a misleading exposure (due to hospitalisation) may have given a larger observed effect. Increased risk may be experienced in community based subjects due to reduced accesses to medical care that may have been able to prevent early symptoms from progressing to a critical phase. The bias associated with earlier recognition, and more timely and aggressive therapeutic intervention may be the reason for the apparently negative %RR associated with an increase in BS of 10 μgm^-3^ within the “non-CDP” only subject group.

This study finds a similar increase in BS exposure effects when compared to U.S. studies of TSP/PM_10_ on all cause mortality when restricted to deaths located outside of hospital [16,17]. Black smoke contains finer particle fractions dominated by combustion emissions which are more closely associated with health outcomes than PM_10_ or PM_2.5_, and so BS filter darkness measurements are a better marker for harmful combustion-related particles [33]. Currently, few published articles comparing indoor and outdoor BS levels exist. Gotschi et al. compared indoor and outdoor BS and PM_2.5_ for 186 homes in Athens, Basel, Helsinki and Prague. The median indoor-outdoor ratios of BS were slightly less than PM_2.5_, however, Spearman correlation coefficients were larger, possibly due to stronger indoor influences on PM_2.5_[34]. Hoek et al. gave filter darkness regressions slopes (0.63-0.84) between indoor-outdoor concentrations in homes of four European cities [35]. Limited information with inconsistent outcomes, often due to small sample sizes (N ≤ 50), is available comparing indoor and outdoor personal particulate matter exposure [36-38]. Janssen N.A.H et al. investigated personal, indoor and outdoor fixed site exposure to PM_10_ in 37 participants from Amsterdam and PM_2.5_ and BS in 36 and 46 participants from Amsterdam and Helsinki respectively. Sampling was taken for 24 hr periods, bi-weekly, over six months. In both cases, median concentrations were found in the personal monitors, followed by outdoor monitoring and then indoor. High correlations were produced between personal and outdoor fixed site monitors indicating that fixed site monitors are a good representation of the day-to-day variation in particulate matter exposure [39,40]. However, high correlation does not imply the same absolute levels. The underlying premise; that exposure to airborne pollutants is reduced in hospitalised subjects; is supported by Wang et al. which showed a reduction in indoor concentrations of PM_10_ and PM_2.5_ in 2 of 4 hospitals in Guangzhou, China [41]. Subsequently, Wang et al. and later Morawska et al. further determined that a mechanical ventilation air conditioning system produced the lowest indoor-outdoor PM_10_ ratios [42,43]. Indoor air quality is an important issue for hospitals. However, currently the quantity of literature available on the relationship between indoor and outdoor air pollution, particularly regarding hospitals, is limited. Further observational studies are required to supplement understanding of the reduction and fluctuations in indoor hospital air pollution concentrations, in terms of distance from combustion sources, changes in ventilation systems and meteorological conditions [44].

One possible alternative explanation for the increase in relative risk occurring within the CDP group is that exposure may have a differing interaction with certain types of pneumonia that are specifically associated with the community. Pneumonia infection can be caused by a variety of micro-organisms. Hospital Acquired Pneumonia is primarily caused by Staphylococcus aureus or Gram-negative enterobacteria, and CAP is most commonly Streptococcus pneumoniae (35% of CAP cases) [45]. Streptococcus pneumonia has an incubation period of 1–3 days, shorter than other pathogens such as Haemophilus influenzae and Mycoplasma pneumonia with incubation periods of 2–4, and 6–32 days respectively [22]. Variation in source and incubation period may be a contributing confounding factor to the difference in BS effect on CDP mortality. Limited information from ICD coding constructed from the death certificate, allowed the pneumonia deaths to be classified into; bronchopneumonia (81%), pneumococcal & streptococcal pneumonia (5%), organism unspecified (13%), and all others (1%), of which 67%, 52%, 47%, and 35%, respectively were CDP subjects. Change in exposure effects on differing underlying causes of pneumonia may be a possible explanation for higher relative risk in CDP deaths. If type of pneumonia was the only explanation for higher RR, then we might expect the RRs for BS within the categories of pneumonia to be the same. In fact, the same pattern of a higher RR for CDP compared to hospital deaths was found for; bronchopneumonia, and pneumococcal & streptococcal types (although not for; organism unspecified and all other types which had much smaller sample sizes reducing the power available to determine the true effect).

However, pollution itself may be the causal factor. Particulate pollution may increase the risk of contracting pneumonia in a number of ways; by impairing microbial clearance via the mucociliary mechanism [46], hindering macrophage phagocytosis [47], or causing intense capillary engorgement and loss of epithelium [48]. These effects might require an indeterminate dose (product of exposure concentration, respiratory minute volume and time) to materialise before manifesting in an increased susceptibility to pneumonia mortality. The temporal relationship between air pollution and pneumonia death analysed here may therefore comprise a period of chemical insult before, as well as a diagnosis to death interval enveloping the 'incubation period’ as classically defined. These varying and relatively indeterminate periods may thus explain why %RR is reduced in the 7–12 day lag compared to 1–6 and 13–18 day lags (Table 2).

A comparison of the BS lag periods within the two models indicated the possible presence of mortality displacement within the data. Mortality displacement, also known as harvesting, is the accelerated progression of a frail sub-population to death followed by a delay in its replenishment. This is thought to be illustrated by an increase in the death rate from its baseline for a certain period after exposure, followed by a period when the death rate seems to be below expected [17]. In Table 2 the lags post 18 days seems to suggest a negative relative risk but in fact this may be due to the shrinking of the at risk population. When the overall effect across the 30 days is estimated (Table 1), the positive and negative estimates balance out to some extent. These results are therefore consistent with the mortality displacement phenomenon. Pneumonia mortality may be more susceptible to 'harvesting’ as pneumonia is prevalent in the elderly [9,49] and is often the final cause in the chain of causes leading to death, implying a high incidence in the frail sub-population compared to the general population. If mortality displacement is present then it would have been easy to miss any risk period if the model only included one term representing average black smoke across 30 days.

The stronger risk experienced for the 18 days prior to death in males within the CDP group may be due to a more outdoors lifestyle in males causing an increased interaction with exposure. Younger subjects may also be expected to experience higher exposure to outdoor pollution concentrations. Yet we found no age group difference in risk within the CDP group possibly due to relatively few subjects aged 65 or less (9.2%), or due to elderly patients being allowed to die at home rather than in hospital. One could further argue that it is difficult to accurately determine the exposure level for these subjects as the area contains only one exposure measurement site. Any exposure misclassification could be reduced if the number of measurement sites could be increased, making it easier to evaluate local variations in pollution levels. Even then, it is difficult to determine a subject location during the entire exposure period, especially when multiple control exposure periods are used. Other than to explore the relationship between BS and mortality; restricting the data to an 18 day lag period was not a part of our original aims and so we do recognise that we lose some validity in our p-values. However, as the 18 day lag period showed significant increases in risk we felt it was worth investigating further.

The study time period (1981–1996) does not necessarily limit these results to a historical interest only given that the primary aim was to compare the effect of pollution on subjects in the community versus within hospital. In fact, the higher outdoor concentrations of black smoke in the 1980's and early 1990's were advantageous for testing this hypothesis given that higher pollution concentrations meant that the incidence of pollution related pneumonia mortality would have been higher and this would make the difference in risk, if any, associated with indoor and outdoor exposure easier to detect. The dominant source of black smoke during both the study period and present day were from road vehicles as most smoke control procedures to reduce combustion of coal for domestic heating and industrial energy would already have been implemented by the start of the study period. However, the chemical composition of fine black particles may have altered somewhat since the study period as a result of technological changes in vehicle engine and emissions control systems. It is not possible to directly characterise the extent of such changes as it is not possible to selectively collect black particles from non-black particles during atmospheric sampling for chemical analyses.

## Conclusions

In conclusion, evidence suggests that a subject’s location is an important factor in relation to their likelihood of pneumonia mortality due to particulate pollution exposure. Including subjects who may have a lower exposure may increase bias in your results and as shown here underestimate the true effect of exposure on pneumonia deaths. The risk to mortality in all subjects, and in particular within the CDP group, tends to last a minimum of 18 days and peaks at the 13–18 day lag. This confirms that air pollution effects do exist beyond short term exposure periods such as 1–3 days, making it is important to investigate extended exposure periods of at least two to three weeks prior to death.

## Abbreviations

AP: All pneumonia; BS: Black smoke; CAP: Community acquired pneumonia; CDP: Community deaths from pneumonia; COPD: Chronic obstructed pulmonary disease; PM10: Particle matter with an aerodynamic diameter less than 10 μgm^-3^; PM2.5: Particle matter with an aerodynamic diameter less than 2.5 μgm^-3^; %RR: Percentage relative risk; TSP: Total suspended particles; VIF: Variance inflation factor.

## Competing interests

The authors declare that they have no competing interests.

## Authors’ contributions

MG performed the statistical analysis and drafted the paper. RM contributed to study idea, its design, statistical analysis and provided reviewers comments. MC helped conceive the study, provided advice, and reviewed the paper. IB contributed to the discussion and reviewed the paper. RA conceived of the study, participated in acquiring the data, provided advice for the discussions and reviewed the paper. All authors read and approved the final manuscript.

## Supplementary Material

Additional file 1: Table S1 Subgroup analysis of 18 day exposure split by subject characteristics; split by AP, CDP & Non-CDP. Click here for file
