# Supplementary material for: Has the short-term effect of black smoke exposure on pneumonia mortality been underestimated because hospitalisation is ignored: findings from a case-crossover study
Source: Environ Health. 2013 Nov 7;12:97. doi: 10.1186/1476-069X-12-97 (PMC4226206; doi:10.1186/1476-069X-12-97)
Supplement: Additional file 1: Table S1 — Subgroup analysis of 18 day exposure split by subject characteristics; split by AP, CDP & Non-CDP. [file 1476-069X-12-97-S1.pdf]

Additional file 1: Table S1 – Subgroup analysis of 18 day exposure split by subject characteristics; split by AP, CDP & Non-CDP

			AP		CDP only		Non-CDP Only				
		Lag (days)	% RR Change	95% C.I	% RR Change	95% C.I	% RR Change	95% C.I	|CDP| – |AP| Diff	|CDP-Non CDP| Diff	p-val
Overall	Black Smoke	1-30	0.30%	0.03%,0.59%	0.70%	0.29%,1.14%	-0.10%	-0.46%,0.30%	0.40%	0.80%	0.001
	Air Temp "Low"	1-30	0.14%	0.03%,0.25%	0.14%	0.00%,0.29%	0.13%	-0.03%,0.29%	0.00%	0.01%	0.141
	Air Temp "High"	1-30	-0.08%	-0.26%,0.11%	-0.20%	-0.45%,0.06%	0.05%	-0.22%,0.33%	0.12%	0.25%	0.221
Male	Black Smoke	1-30	0.32%	-0.09%,0.75%	0.83%	0.21%,1.51%	-0.14%	-0.66%,0.45%	0.51%	0.97%	0.015
	Air Temp "Low"	1-30	0.18%	0.02%,0.34%	0.16%	-0.05%,0.38%	0.18%	-0.05%,0.42%	0.02%	0.02%	0.442
	Air Temp "High"	1-30	-0.15%	-0.41%,0.13%	-0.40%	-0.76%,-0.01%	0.12%	-0.28%,0.54%	0.25%	0.52%	0.067
Female	Black Smoke	1-30	0.30%	-0.07%,0.69%	0.61%	0.08%,1.19%	-0.07%	-0.56%,0.47%	0.32%	0.68%	0.029
	Air Temp "Low"	1-30	0.11%	-0.03%,0.25%	0.12%	-0.07%,0.32%	0.08%	-0.13%,0.29%	0.02%	0.04%	0.188
	Air Temp "High"	1-30	-0.02%	-0.27%,0.24%	-0.04%	-0.38%,0.33%	-0.01%	-0.37%,0.37%	0.02%	0.03%	0.992
Age <80	Black Smoke	1-30	0.23%	-0.17%,0.66%	0.71%	0.10%,1.38%	-0.19%	-0.71%,0.38%	0.47%	0.90%	0.058
	Air Temp "Low"	1-30	0.11%	-0.06%,0.27%	0.03%	-0.19%,0.26%	0.17%	-0.06%,0.41%	0.08%	0.14%	0.929
	Air Temp "High"	1-30	-0.05%	-0.32%,0.24%	-0.30%	-0.67%,0.11%	0.19%	-0.20%,0.61%	0.25%	0.49%	0.065
Age ≥80	Black Smoke	1-30	0.37%	0.00%,0.77%	0.71%	0.18%,1.30%	-0.02%	-0.52%,0.53%	0.34%	0.73%	0.009
	Air Temp "Low"	1-30	0.16%	0.03%,0.31%	0.22%	0.03%,0.41%	0.09%	-0.11%,0.31%	0.05%	0.13%	0.005
	Air Temp "High"	1-30	-0.10%	-0.34%,0.16%	-0.13%	-0.45%,0.22%	-0.09%	-0.45%,0.30%	0.03%	0.04%	0.963

%RR Change - percentage change in Relative Risk, associated with an increase of 10μgm^{-3} BS or a decrease of 1°C, on any individual day within the lag period, with corresponding 95% Confidence Interval (95% C.I.)

Model 1 - One 30 day lag, Model 2 - The 30 days split into 5 lags of 6 days each fitted simultaneously

|CDP| – |AP| Diff - The difference in the magnitude of the effect size between AP and CDP (CDP-AP)

|CDP-Non CDP| Diff - The difference in the effect size between CDP and Non-CDP
